# Ecological and biogeographic features shaped the complex evolutionary history of an iconic apex predator (*Galeocerdo cuvier*)

**DOI:** 10.1186/s12862-022-02100-y

**Published:** 2022-12-16

**Authors:** Pierre Lesturgie, Hugo Lainé, Arnaud Suwalski, Pascaline Chifflet-Belle, Pierpaolo Maisano Delser, Eric Clua, Sébastien Jaquemet, Hélène Magalon, Stefano Mona

**Affiliations:** 1 Institut de Systématique, Evolution, Biodiversité (ISYEB), EPHE-PSL, Université PSL, MNHN, CNRS, SU, UA, Paris, France; 2 EPHE, PSL Research University, Paris, France; 3 Department of Zoology, University of Cambridge, Cambridge, UK; 4 Laboratoire d’Excellence CORAIL, Papetoai, French Polynesia; 5 UMR ENTROPIE (Université de La Réunion/IRD/CNRS), Université de La Réunion, Saint Denis, France

**Keywords:** Agulhas leakage, Coalescent modelling, Demographic history, Population genomics, RAD-seq, Tiger shark

## Abstract

**Background:**

The tiger shark (*Galeocerdo cuvier*) is a large iconic marine predator inhabiting worldwide tropical and subtropical waters. So far, only mitochondrial markers and microsatellites studies have investigated its worldwide historical demography with inconclusive outcomes. Here, we assessed for the first time the genomic variability of tiger shark based on RAD-seq data for 50 individuals from five sampling sites in the Indo-Pacific (IP) and one in the Atlantic Ocean (AO) to decipher the extent of the species’ global connectivity and its demographic history.

**Results:**

Clustering algorithms (PCA and NMF), *F*_*ST*_ and an approximate Bayesian computation framework revealed the presence of two clusters corresponding to the two oceanic basins. By modelling the two-dimensional site frequency spectrum, we tested alternative isolation/migration scenarios between these two identified populations. We found the highest support for a divergence time between the two ocean basins of ~ 193,000 years before present (B.P) and an ongoing but limited asymmetric migration ~ 176 times larger from the IP to the AO (*Nm* ~ 3.9) than vice versa (*Nm* ~ 0.02).

**Conclusions:**

The two oceanic regions are isolated by a strong barrier to dispersal more permeable from the IP to the AO through the Agulhas leakage. We finally emphasized contrasting recent demographic histories for the two regions, with the IP characterized by a recent bottleneck around 2000 years B.P. and the AO by an expansion starting 6000 years B.P. The large differentiation between the two oceanic regions and the absence of population structure within each ocean basin highlight the need for two large management units and call for future conservation programs at the oceanic rather than local scale, particularly in the Indo-Pacific where the population is declining.

**Supplementary Information:**

The online version contains supplementary material available at 10.1186/s12862-022-02100-y.

## Background

Predation plays a fundamental role in the top-down regulation of ecosystem dynamics, with apex predators being key actors in promoting species diversity [1]. However, in marine ecosystems, many predatory species have declined across their ranges [2]. Efforts to develop conservation programs need be tailored to the appropriately scaled units of managements for the species under investigation [3]. Recent advances in DNA sequencing technologies allow the characterization of thousands of independent loci giving the power to assess the genetic diversity of any target model or non-model species, which can inform management policies. However, genetic diversity assessment should be complemented by the reconstruction of species connectivity and historical demography to better establish conservation priorities. For instance, understanding how populations are spatially connected as well as the divergence time between lineages is essential to decipher at which geographic scale a species should be managed. Reconstructing the evolutionary history of a species is often a complex task that requires an educated choice of the most likely model of evolution, often selected among a reduced selection of biologically meaningful models. Unfortunately, selection of an inappropriate model can yield misleading estimates of critically important parameters as more data are collected. This has important implications for conservation genetic applications that rely on accurate estimates of genetic diversity and changes in effective population size through time [4–7].

The tiger shark (*Galeocerdo cuvier,* Péron & Lesueur, 1822) is a large and iconic apex marine predator, that is considered “Near Threatened” by the International Union for Conservation of Nature (IUCN). Though the tiger shark is a predominantly coastal species, its distribution includes tropical and subtropical waters worldwide [8]. The species is heavily impacted by fisheries [9] and shark control programs in the Indo-Pacific [10]. Indirect estimates have suggested an annual number of tiger shark catches between 50,000 and 300,000 individuals [11], raising conservation concerns. Though not directly endangered by global climate change, the species is likely to extend its habitat range poleward as a consequence of the rising in annual sea surface temperatures [12], which may increase the potential for greater trans-oceanic movements [13]. Despite being found predominantly along the coast, tiger sharks spend considerable time in pelagic waters and telemetry studies have shown that they can cross oceanic expanses [14–16], but no evidence of contemporary migration between the Indo-Pacific and the Atlantic Ocean has yet been found.

**Fig. 1:**
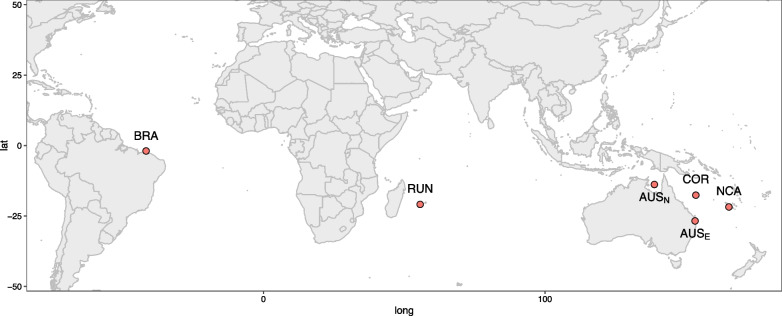
Map of the sampling sites. From west to east: Brazil (BRA: n = 7), Reunion Island (RUN; n = 15), North Coast of Australia (AUS_N_; n = 8), Coral Sea (COR; n = 5), East Coast of Australia (AUS_E_; n = 7) and New Caledonia (NCA; n = 8)

Knowledge of these ecological traits is important to devise meaningful evolutionary models, but it is not sufficient. Large marine predators with continuous distributions can be intuitively considered as capable of high dispersal due to the absence of clear physical barriers [17]. Nevertheless, there are both examples of panmictic species, such as the blue shark *Prionace glauca* [18] or the mako shark *Isurus oxyrinchus* [19], and examples of species structured according to ocean basins such as the Galapagos shark *Carcharhinus galapagensis* and the dusky shark *Carcharhinus obscurus* [20]*.* In the tiger shark, there has been contrasting evidences about the degree of population structure, the extent of genetic diversity and particularly about the historical demography [21–27]. All studies recognize the existence of a clear separation between the Indo-Pacific (IP) and the Atlantic Ocean (AO), with genome-wide data supporting low to no population structure within each basin [27]. However, it remains unclear whether the two basins hold two allopatric species as originally proposed by Naylor et. al (2012) or two divergent lineages as proposed by Bernard et. al (2016). An accurate characterization of divergence and migration is additionally still lacking: Bernard et al. (2016) provided a divergence time computed on mtDNA using a non-equilibrium model implemented in mdiv [28] but no confidence interval could be determined. At the same time, migration rates were estimated using the equilibrium model implemented in Migrate [29]*.* Yet a global analysis estimating simultaneously all parameters is warranted. Furthermore, an in-depth analysis of the historical demography in both regions is still lacking, with only [24] supporting a recent decrease in effective population size in two sampling sites from the IP. Using the wealth of data provided by RAD-seq, we sequenced a total of 50 sharks from six sites in the IP and one in the AO (Fig. 1) in order to shed light on the complex evolutionary history of the tiger shark. We first investigate the extent of genetic diversity, the level of population structure and historical demography in all sampling sites, and finally tested alternative evolutionary scenarios to model the divergence and migration between IP and AO by fitting the observed two-dimensional site frequency spectrum (2D-SFS) with coalescent simulations. These analyses are necessary not only to reconstruct the evolutionary history of the tiger shark but also to better inform conservation strategies.

## Results

### RAD-seq sequencing

The average number of reads retained per individual after the quality filtering and demultiplexing step was 4,011,430 (± 1,314,894 s.d.). After a first round of de novo assembly and filtering using STACKS v.2.5, the depth of coverage was low with a mean of 12.65 (± 6.36 s.d.), which motivated the use of the genotype-free allele frequency estimation pipeline implemented in angsd [30] rather than the direct call. The final number of loci (variable and fixed) was highly variable between sampling locations (Table 1) ranging from 16,953 to 118,591 in Brazil (BRA) and New Caledonia (NCA) sampling sites respectively, with a number of SNPs with no missing data following a similar trend (from 5868 to 26,075 for BRA and NCA, respectively).

**Table 1:** Sample size (n), mean pairwise difference (θ_π_), Watterson theta (θ_w_), Tajima’s D (TD), and total number of loci (monomorphic included) (n_loci_) and SNPs (n_SNP_) without missing data for all sampling sites (ranged from west to east)

	n	θ_π_ (10^–3^)	θ_w_ (10^–3^)	TD^a^	n_loci_	n_SNP_
BRA	7	0.97	1.16	**0.14**	16,953	5868
RUN	15	0.59	0.73	**− 0.19**	71,214	19,971
AUS_N_	8	0.76	0.97	**− 0.19**	38,420	11,627
COR	5	0.58	0.72	0.04	97,736	18,407
AUS_E_	7	0.63	0.77	0.02	49,380	11,385
NCA	8	0.57	0.7	**− **0.03	118,591	26,075

*AUS*_*E*_ East Coast of Australia, *AUS*_*N*_ North Coast of Australia, *BRA* Brazil, *COR* Coral Sea, *NCA* New Caledonia, *RUN* Reunion Island

^a^Tajima’s *D* values in bold are significantly different from 0 (*P* < 0.001)

### Population structure

**Fig. 2:**
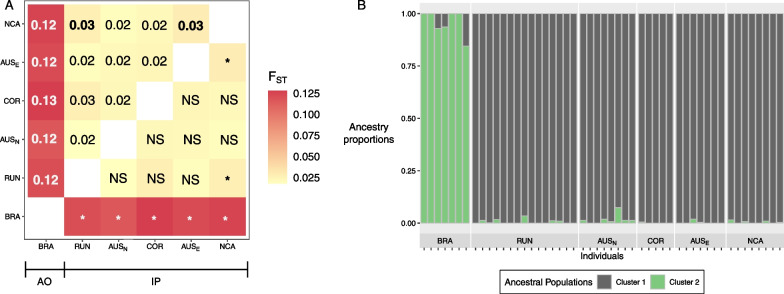
Heat map representing the pairwise Reynold’s *F*_*ST*_ values between sampling sites (**A**) and ancestry proportions retrieved using the *nmf* algorithm with K = 2 ancestral populations (**B**). Both analyses were performed with PCAngsd. Values in the upper triangle of the heat map are the pairwise FST values and significance is displayed on the lower triangle: non-significant (NS) or p < 0.001 (*)

**Fig. 3:**
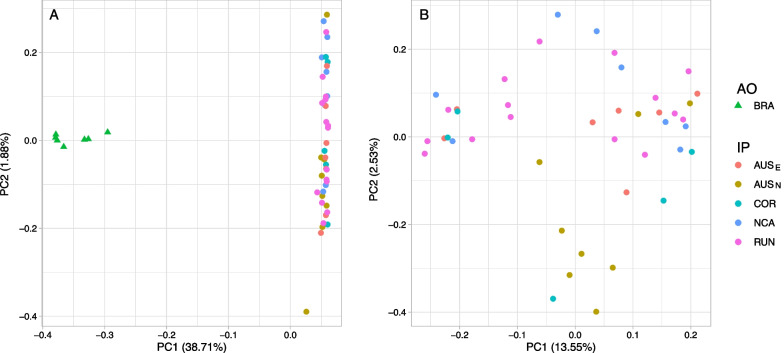
Principal Component Analysis (PCA) computed with: (**A**) all individuals (n = 50) and (**B**) Indo-Pacific individuals only (n = 43)

Population structure was investigated using datasets allowing up to 20% of missing data per SNP. Thus, after filtering, the remaining number of SNP was 24,454 for the Principal Component Analysis (PCA) and the *non-negative matrix factorization* (*nmf*) inference, and ranged from 8785 to 15,977 per population pair for the *F*_*ST*_ computation. Pairwise *F*_*ST*_ highlighted a moderate differentiation between Indo-Pacific (IP) and Atlantic Ocean (AO) sampling sites, with values ranging from 0.117 to 0.129 and systematically significant (*P* ≤ 0.001, Fig. 2A and Additional file 1: Table S1). Conversely, the average *F*_*ST*_ between IP sites was 0.023 (ranging from 0.018 to 0.029) and not statistically significant for the majority of pairwise comparisons (Additional file 1: Table S1). The Mantel test, computed between IP sampling sites only (given the evidence of a clear genetic discontinuity between AO and IP), showed no correlation between genetic and geographic distances (r = 0.005, *P* = 0.62, Additional file 1: Fig. S1). Clustering analyses were consistent with the observed pattern of differentiation. First, the *nmf* algorithm selected *K* = 2 ancestral populations corresponding to IP and AO (Fig. 2). Individuals from IP had a probability ancestry to cluster 1 ranging from 92.6 to 100% whereas individuals from AO showed a probability ancestry to cluster 2 ranging from 84.6 to 100%. Average ancestry of cluster 2 in IP individuals was only 0.7% while average ancestry of cluster 1 in AO individuals was 4.4%. Second, the PCA clearly segregated AO from IP individuals, with 38.71% of the total variance explained by the first axis (Fig. 3A). Individuals from Reunion Island (RUN), the IP site closest to the AO, did not show more proximity to the AO in the PCA or a higher contribution from cluster 2 than other IP individuals, nor did they show a lower pairwise *F*_*ST*_ with the AO than the other IP sites. (Figs. 2, 3A and Additional file 1: Fig. S2A). When computed on IP individuals only, the PCA identified a single cluster (Fig. 3B and Additional file 1: Fig. S2B) and the *nmf* did not show any meaningful geographic clusters with *K* = *2* (Additional file 1: Fig. S3). We further applied an Approximate Bayesian Computation (ABC) framework using a 500 trees random forest for all sampling sites after checking for the evolution of the out-of-bag error rate. This coalescent framework allows to detect genetic structure using a single sampling location by testing whether its gene genealogy yield signatures of a Stepping Stone (SS), Finite Island (FIM) or Non-Structured (NS) model (Additional file 1: Fig. S4). The model selection (Additional file 1: Table S2) highlighted NS as the most supported model with a posterior probability ranging from 0.48 to 0.89 in the IP sampling sites and of 0.62 for BRA (Additional file 1: Table S3).

### Genetic diversity and variation of *N*_*e*_

**Fig. 4:**
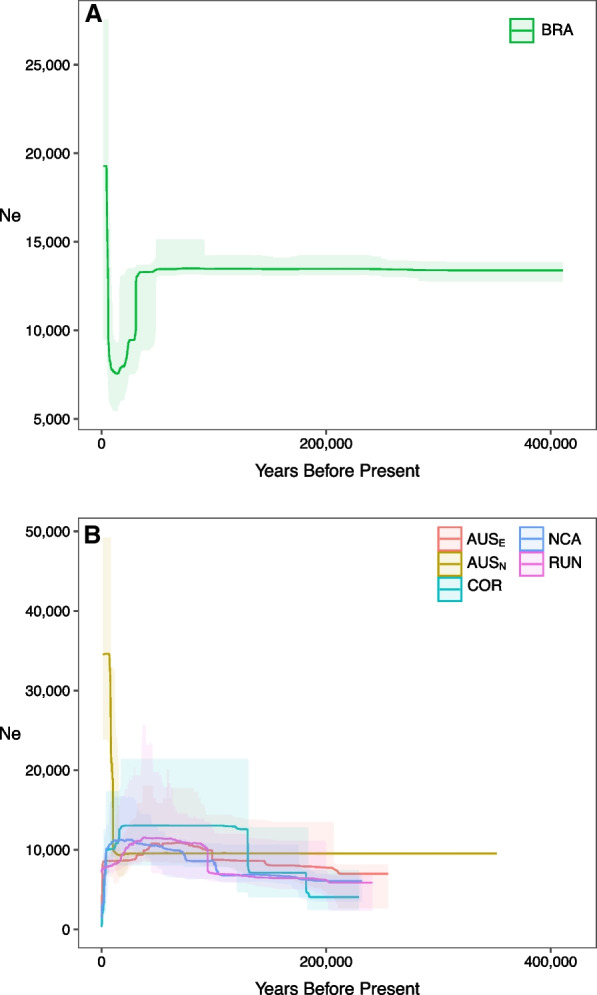
Variation of the effective population size (*N*_*e*_) through time and its 75% confidence interval estimated by the stairwayplot for the AO (panel **A**) and IP (panel **B**) sampling sites. *AUS*_*E*_ East Coast of Australia, *AUS*_*N*_ North Coast of Australia, *BRA* Brazil, *COR* Coral Sea, *NCA* New Caledonia, *RUN* Reunion Island

Genetic diversity values were very similar among sampling sites, with BRA being slightly more variable than the IP counterpart (Table 1). Tajima’s D (*TD*) was significantly positive for BRA (*TD* = 0.137; *P* ≤ 0.001), while significantly negative (*P* ≤ 0.001) for Northern Australia (AUS_N_) and Reunion Island (RUN) and not significantly different from zero for the other populations (Table 1). Except for the AUS_N_ population, the reconstructions of the effective size (*N*_*e*_) through time by the stairwayplot were very similar among IP locations: an ancestral expansion occurred between ~ 100,000 and ~ 200,000 years before present (BP). bringing the median *N*_*e*_ to ~ 10,000 followed by a very recent bottleneck ~ 2000 to ~ 4000 years BP (Fig. 4). The stairwayplot for AUS_N_ displayed a different signal, with an ancestral *N*_*e*_ median value similar to the one retrieved in the other sampling sites (~ 10,000) followed by a strong and recent expansion that raised the modern *N*_*e*_ to ~ 35,000, contrasting with the recent decrease observed for the other IP sampling sites. The demographic history reconstructed for BRA was slightly more complex with the ancestral *N*_*e*_ of ~ 12,000 first decreasing to ~ 9000 at ~ 40,000 years BP and then increasing (between ~ 4000 and 6000 years BP) to a modern *N*_*e*_ of ~ 20,000 (Fig. 4).

### Population divergence and migration rate estimation

**Fig. 5:**
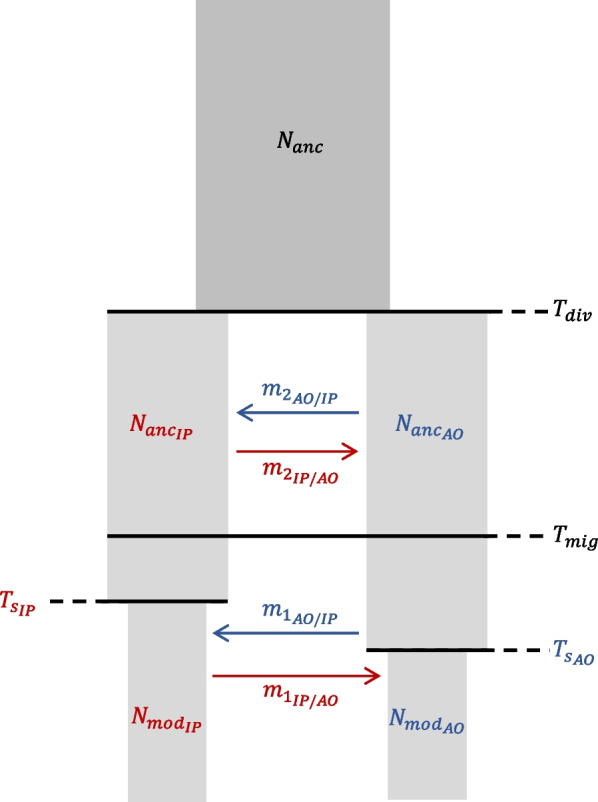
Model IM-full, the most parameter-rich model (13 parameters) representing two populations from each ocean basin with an effective size that changed ${T}_{{s}_{IP}}$ and ${T}_{{s}_{AO}}$ years ago from a modern effective size (${N}_{{mod}_{IP}}$ and ${N}_{{mod}_{AO}}$) to an ancestral effective size (${N}_{{anc}_{IP}}$ and ${N}_{{anc}_{AO}}$). The two populations are connected by an asymmetrical migration rate allowed to change ${T}_{mig}$ years ago (respectively from ${m}_{{1}_{IP/AO}}$ and ${m}_{{1}_{AO/IP}}$ to ${m}_{{2}_{IP/AO}}$ and ${m}_{{2}_{AO/IP}}$) and diverged ${T}_{div}$ years ago from an ancestral population of size ${N}_{anc}$. The remaining four models are nested within IM-full, having less migration rate parameters: IM-anc is similar to IM-full but only the ancestral migration occurs (i.e., between ${T}_{mig}$ and ${T}_{div}$); IM-rec is similar to IM-full but only the recent migration occurs (i.e., between 0 and ${T}_{mig}$); IM-bsc considers the migration constant from 0 to ${T}_{div}$; and IM-div is a strict divergence model with no migration

In the light of the absence of population structure signatures within the IP and the AO and the genetic distinctness between them, we tested five Isolation-Migration (IM) models to determine the divergence time and the migration pattern between the two oceanic regions (Fig. 5). The likelihood distribution computed over 100 replicates was similar for models IM-bsc and IM-full, but the AIC values supported IM-bsc as the model with the highest probability (Additional file 1: Fig. S5). The distribution of the likelihood evaluated in each model under the Maximum Likelihood (ML) parameters proved that they can be distinguished based on the available data (Additional file 1: Fig. S5). The two oceanic regions appeared connected, though the migration rate is limited and strongly asymmetric, being ~ 176 times higher from IP to AO (*Nm* ~ 3.9) than vice versa (*Nm* ~ 0.02) (Table 2 and Additional file 1: Fig. S6). Going backward in time the populations from the two regions merged ~ 193,000 years BP (90% CI: [77,000; 355,000]). The ancestral population size was almost half of those estimated in both IP and AO derived populations (Table 2 and Additional file 1: Fig. S6), indicating an ancestral expansion, consistent with the observed stairwayplot dynamics for IP populations. An increase in effective size was estimated in the AO starting ~ 45,000 years BP (90% CI: [14,000; 53,000]), bringing the effective size from 1,406 (90% CI: [1,178; 4,781]) to 16,810 (90% CI: [12,885; 40,036]) which is consistent with the observed expansion in the stairwayplot of BRA. We observed a decrease in *N*_*e*_ in the IP from 49,000 to 17,000, but the timing was poorly estimated. Moreover, ancient and modern *N*_*e*_ in the IP showed largely overlapping confidence intervals (Table 2).

**Table 2:** Maximum Likelihood (ML), 90% confidence interval (5% lower bound and 95% upper bound) and search bounds for the parameters estimated by fastsimcoal under the IM-bsc model

Parameter	ML	5% Lower bound	95% Upper bound	Parameter bounds
${N}_{anc}$	9274	1310	80,372	U:{100;100,000}
${N}_{mo{d}_{IP}}$	17,591	16,643	31,915	U:{100;100,000}
${N}_{mo{d}_{AO}}$	16,810	12,885	40,036	U:{100;100,000}
${T}_{{s}_{IP}}$ a	153,090	27,460	769,750	U:{100;1,000,000}
${T}_{{s}_{AO}}$ a	45,980	14,030	53,090	U:{100;1,000,000}
${N}_{{anc}_{IP}}$	48,823	3103	107,816	U:{100;100,000}
${N}_{{anc}_{AO}}$	1406	1178	4781	U:{100;100,000}
${T}_{div}$ a	193,850	77,110	355,910	U:{100;1,000,000}
${Nm}_{AO/IP}$_b_	0.022	0.008	0.124	U:{0;50}
${Nm}_{IP/AO}$ ^b^	3.873	3.171	8.919	U:{0;50}

^a^Times are expressed in years using a mutation rate of 1.93 × 10^–8^ per generation per site and a generation time of 10 years

^b^Number of migrants per generation are expressed in forward

*U* uniform distribution

## Discussion

To shed light on the evolutionary history of the tiger shark, we sequenced thousands of loci in 50 individuals following a double digest RAD-seq protocol. We handled low coverage issues by applying an appropriate framework based on genotype free likelihood estimation of allele frequencies [30]. The first result is the unambiguous presence of two highly divergent genetic clusters, corresponding to the Indo-Pacific (IP) region and the Atlantic Ocean (AO), and the signature of very weak population structure within each of them. Despite the large panel of SNPs that could potentially detect fine spatial structure compared to previous work based on microsatellites, we did not find any barrier to gene flow within the IP, but rather a signature consistent with a large panmictic population or a meta-population characterized by very large amount of gene flow (Figs. 2, 3, Additional file 1: Figs. S1, S2, S3). This strongly confirms previous findings [23, 24, 27] and contradicts the conclusions of [22], who found significant evidence for population structure. Using a larger amount of genomic information, we provide evidence for the presence of a single mating population in the IP based on the following observations: (1) the PCA and *nmf* analyses display one single cluster in the IP; (2) the *F*_*ST*_ values computed between sampling sites did not exceed 0.029 (as a comparison, an average value of ~ 0.124 was found between IP and AO) with no signature of isolation by distance (Additional file 1: Fig. S1); (3) all IP sharks showed a similar amount of genetic contribution from the AO cluster in the *nmf* analysis (Fig. 2). These results are consistent with one of two explanations: either (1) tiger sharks randomly mate within IP or (2) the number of migrants exchanged each generation between sampling sites is so large to erase any signature of genetic structure. The absence of multiple sampling sites in AO prevented us to perform similar analyses in AO. To test the presence of a single panmictic population, we therefore followed an ABC strategy based on coalescence simulations to reconstruct the evolutionary history of BRA population and assess whether the patterns of genetic variation within the BRA samples are better described by unstructured or meta-population models. This approach has been successfully applied in both empirical and simulation-based works [7, 31, 32] and represents an alternative (or complementary, when possible) method to infer the presence of population structure. As found for the IP, the NS (No Structure) model had the most support within the AO (Additional file 1: Table S3). Even though more sampling sites and SNPs would likely have provided tighter estimates, this result is most consistent with a single mating population in the AO, despite our single sampling site does not allow us to infer the geographic extent of that population. We note that population structure has previously been reported within the AO based on mitochondrial markers [25, 26]. However, results in the current study are consistent with the genome wide study of [27] which suggested low to no population structure in the AO. The differences between the inferred mitochondrial structure and genomic DNA signals could be due to sex-biased dispersal, as female philopatry has been proposed for some shark species [33–36]. However, inconsistencies between mitochondrial and autosomal data are widespread in nature and it has been suggested to cautiously interpret the mitochondrial variation in the light of the demographic history of a species, as other evolutionary forces such selection may act as confounding factors [37]. Furthermore, the result found herein (i.e., low to no population structure in each of the two oceanic regions) is consistent with the fact that tiger sharks have been documented to move large distances across oceanic basins. However, it does not explain why a large predator that is capable of covering distances of several thousands of kilometers [13–15] could not erase the genetic differentiation between the two regions.

One possible explanation was proposed by [21], who suggested the presence of two allopatric subspecies in IP and AO. This hypothesis was later refuted by [22], who still agreed on a long-term genetic isolation between the two oceanic regions but proposed some genetic exchanges. By harnessing the power of RAD-seq genome wide data, our coalescent modelling could not only disentangle the two hypotheses, but also provide quantitative estimates of the tempo and mode of divergence between the two populations. Comparing five Isolation/Migration (IM) scenarios, we found that the most supported model included a divergence around ~ 193,000 years BP (90% CI: [77,000; 356,000]) between the two regions (Table 2), which nevertheless remained in contact since then through a very limited (3.9 individuals per generation; 90% CI: [3.2; 8.9]) and asymmetric gene flow ~ 176 times higher from IP to AO than the opposite direction. The low number of migrants *Nm* exchanged each generation (Table 2) and the asymmetric exchange are consistent with the clustering results and the *F*_*ST*_ values (Fig. 2), which differentiated the two regions and clearly highlighted a higher, but weak, genetic contribution from IP to AO than vice versa (Fig. 2). These results support the idea that populations from the AO and from the IP represent two lineages [22], rather than two allopatric species [21]. A permeable barrier to gene flow between the two oceanic regions is therefore responsible for the observed pattern of divergence and asymmetric migration. The presence of this barrier can be explained by the ecology of the tiger shark and by the environmental conditions governing the Indian-Atlantic water exchange, the so-called Agulhas leakage. As a tropical to sub-tropical species, tiger sharks prefer warm water and they show the peak of swimming activities at ~ 22 °C [12] so that their movement from the Atlantic to the Indo-Pacific is hampered by the upwelling of cold water off South-Western Africa (the Benguela current). However, the AO receives warm water from the IP through the Agulhas leakage [38], which can account for the asymmetric migration reported, consistent with the pattern observed in other tropical sharks, bony fishes and turtles [39–42]. The Agulhas leakage has not been constant through time, with an increasing intensity in the Holocene, preceded by a period of stasis and a strong peak in the late Pleistocene around 130,000 years BP [43, 44]. This variation in Agulhas leakage intensity could have influenced the relation between the two basins. However, we could not distinguish pulses of migrations in our data since the model IM-bsc was preferred to those accounting for variation in migration rate through time. Model selection procedure robustly selected the IM-bsc model (Additional file 1: Fig. S5), which is neither the most nor the least parameters rich, supporting the idea that our results are not an artefact of incorrect modelling (see also below). In the future there will still be space to improve our estimates: more data will help refining the confidence interval of each parameter and ameliorating the calibration of the molecular clock.

We found divergent demographic histories in the two oceanic regions examined (Fig. 4). First, we note that since both regions are most likely described by non-structured models and the migration rates between them are very low, it is possible to directly interpret the results of unstructured model such as the stairwayplot [45]. It is important to stress this point, because population structure, if not accounted for, can generate signatures that erroneously look like changes in effective population size [32, 46, 47]. All the Indo-Pacific populations (except AUS_N_) underwent a recent bottleneck between ~ 2000 and 4000 years BP, which was robust to the pooling of all IP sampling points but AUS_N_ (but with larger incertitude in recent times due to the lower number of SNPs, see Additional file 1: Fig. S7). Despite the caveat regarding the interpretation of *N*_*e*_ variation in recent times due to the inclusion of singletons, this is barely consistent with what was recently proposed by [24] based on 25 microsatellites combined with mitochondrial DNA. Here we refined their estimates and better characterized the intensity of the bottleneck with a non-parametric approach (the stairwayplot) exploring a large parameter space with genome wide data. The inferred demographic history of the AO is strikingly different from that of the IP. The estimated *N*_*e*_ was ~ 20,000 following a population expansion occurring between ~ 4000  and ~ 6000  years BP (Fig. 4). These values are consistent with the estimates retrieved by the IM-bsc model (Table 2). The strong signature of population expansion recovered implies that the tiger shark is profiting from recent environmental changes in AO, in contrast to the IP population. Consistently, [26, 48] found a recent demographic trend suggesting an expansion rather than a decrease in AO, while the recent demographic trends in the IP appear to have been the result of intense pressure from fisheries and shark-control programs [9–11]. More investigations are needed to determine the origin of the difference between the two oceans. The applications of methods based on linkage disequilibrium applied to whole genome data will help detect more recent events [49], which will be important for planning conservation strategies. Given the very low genetic structure within IP, we would expect AUS_N_ to have the same demographic history than the other sampling sites in the IP. We cannot exclude a scenario where AUS_N_ represents an isolated population experiencing its own demographic history that separated from the rest of the IP too recently to accumulate divergence. If confirmed, this would highlight the presence of independent lineages in the IP, with important consequence for conservation programs. However, more data is needed to shed light on this topic, both in terms of individuals and in terms of genomic coverage: ultimately only whole genome sequencing will give the opportunity to confidently resolve this issue.

## Conclusions

Reconstructing the evolutionary history of a species relies on the application of a realistic demographic model, which is mostly unknown in empirical studies. Here, we investigated the evolutionary history of the tiger shark and found that it is characterized by an asymmetrical migration between the AO and the IP and a signature of random mating within each region. These findings let us model each oceanic region as a single unstructured population and evaluate competing demographic scenarios to investigate their divergence time and migration rates. The two regions are separated by the Benguela barrier, but our estimates strongly suggest that the Agulhas leakage allows an overwhelmingly asymmetric migration between them, by far stronger from IP to AO than in the opposite direction. While we confirmed that the tiger shark is likely undergoing a reduction in *Ne* in the Indo-Pacific, we show that it probably underwent a strong expansion in the Atlantic Ocean. Even if a better calibration of the molecular clock and full genome analyses would still be needed to confirm our results, our findings support the existence of two management units. This implies that local conservation or shark control programs will have very limited impact on the dynamics of the species, which needs to be managed at the ocean basin level, demanding considerable communication efforts among different countries and coordination as suggested for other megafaunal organisms [50].

## Material and methods

### Sampling, library preparation and sequencing

A total of 50 tiger shark individuals (*Galeocerdo cuvier*) from both the Indo-Pacific (IP) and the Atlantic Ocean (AO) were sampled off (from west to east) Brazil (BRA), Reunion Island (RUN), North Coast of Australia (AUS_N_; North Territory), East Cost of Australia (AUS_E_; Sunshine Coast), Coral Sea (COR) and New Caledonia (NCA). Sharks were grouped into six populations based on their sampling site (Fig. 1; Table 1). Total genomic DNA was extracted from muscle tissue or fin clips preserved in 96% ethanol using QIAGEN DNeasy Blood and Tissue kit (Qiagen, Hilden, Germany) according to the manufacturer's protocols. Double-digest restriction-associated DNA (ddRAD) libraries were prepared following [51] using EcoRI and MspI restriction enzymes, a 400-bp size selection, and a combination of two indexes and 24 barcodes to pool 48 individuals per lane. The genomic libraries obtained were sequenced with a HiSeq 2500 Illumina sequencer (single-end, 125 bp).

### RAD-seq de novo assembly

Raw reads were first demultiplexed and quality filtered through the *process_radtags.pl* pipeline in stacks v.2.5 [52]. In the absence of a reference genome of *G. cuvier* or of closely related species, RAD-seq loci (125 bp sequences) were de novo assembled under the *denovo_map.pl* pipeline in stacks. Preliminary results based on parameters *m* = *3* (minimum read depth to create a stack), *M* = *3* (number of mismatches allowed between loci within individuals), and *n* = *3* (number of mismatches allowed between loci within catalogue) found an average depth of ~ 10x (see Results). Despite the absence of a clear cut-off indicating an acceptable coverage value above which genotype calling may be considered reliable, simulation results suggest that ~ 10x  may produce inconsistent calling under different algorithms [53]. To prevent this, we used a genotype free estimation of allele frequencies implemented in the software angsd v.0.923 (Analysis of Next Generation Sequencing Data; [30]), which has been proven to be a more efficient method for low to medium coverage next-generation sequencing (NGS) data than SNPs calling algorithms [30]. We describe below the bioinformatics steps required to apply angsd to RAD-seq data from a non-model organism and the downstream population genetic analyses applied to the filtered datasets.

### Assembly pipeline and filtering

Angsd requires a reference sequence to work, which we were lacking. To circumvent this issue, we followed the approach described in [54] by creating an artificial reference sequence from loci previously assembled by stacks under the parameter *m* = 3, *N* = 3, *M* = 3 (based on the results of Mona, Bertorelle, Benazzo, in preparation). To this end, we concatenated the consensus sequences of each locus spaced by a stretch of Ns and then map reads back from individual *fastq* files using the *bwa-mem* algorithm with default parameters [55]. Using custom bash scripts coupled with angsd, we then discarded: (i) sites with coverage < 3x (-*minIndDepth* = 3, corresponding to *m* in the first assembly performed by stacks) and/or of low quality (based on the per base alignment score, -*baq* = *1* flag); (ii) low quality bases and poorly aligned reads (-*minQ* and -*minMapQ* and *-C* flags with default values); (iii) SNPs present in the last 5 bp of each locus and SNPs genotyped as heterozygous in 80% or more of the individuals; (iv) loci with more than 5 SNPs that might be the result of paralog RAD loci alignment on the reference. Specific filters were further added according to the downstream analyses performed.

### Population structure

A single reference sequence was created for all populations and we retained sites shared by at least 80% of the samples. The PCA was computed with PCAngsd v.0.97 based on genotype likelihood [56]. Admixture was then investigated by running the *non-negative matrix factorization* algorithm (*nmf*) implemented in PCAngsd which is based on the same covariance matrix inferred for the PCA. The number of ancestral populations (*K*) was automatically chosen by PCAngsd to be *e* + 1, where *e* is the optimal number of significant principal components depicting population structure, resulting from the Velicier’s minimum average partial test run on the covariance matrix. The sparseness regularization parameter *α* (used to reduce the noise in low depth NGS data) that best fitted the data was tested between 0 and 100 and it was chosen by comparing the resulting likelihood following [56]. We generated pairwise *site allele frequency likelihood* files and then computed *F*_*ST*_ with the realSFS program in angsd [57] using SNPs with a minor allele frequency ≥ 0.05 (*-minMaf* flag). The significance of each pairwise *F*_*ST*_ comparison was evaluated with 1000 permutations by randomly allocating individuals to one of the two populations. We finally tested isolation by distance (IBD) using a Mantel test [58] and plotted the relationship between genetic *vs.* geographic distances.

We applied an *approximate Bayesian computation* (ABC) approach similar to previous studies [7, 31, 32] in all sampling sites to further investigate the presence of population structure. This approach is particularly helpful in the Atlantic Ocean (AO) where only one locality was sampled (Brazil; BRA population). Briefly, we designed three demographic models (Additional file 1: Fig. S4): (1) NS (No Structure) which represents a panmictic population where ${N}_{mod}$, the modern effective size instantly changes to ${N}_{anc}$, the ancestral effective size, at ${T}_{s}$(time shift) generations; (2) FIM (Finite Island Meta-population) which represents a finite island meta-population model composed of 100 demes exchanging symmetrically $Nm$ migrants per generation with each other. All demes were instantaneously colonised, ${T}_{col}$ generations ago, from an ancestral population of size ${N}_{anc}$; (3) SS (Stepping-Stone) which represents a stepping-stone model where the 100 demes are arranged in a two-dimensional grid and where migration is only allowed symmetrically in both directions between the four nearest neighbouring demes. We performed 50,000 coalescent simulations under each model using fastsimcoal v.2.6.0.3 [59] extracting parameters from the prior distributions displayed in Additional file 1: Table S1. Model selection was evaluated by the random forest classification method implemented in the *abcRF* package in R [60]. We used the SFS, θ_π_ and *TD* as summary statistics and further added the first two axes of the Linear Discriminant Analysis in the dataset as suggested by [60] to increase the classification method accuracy. The number of trees was chosen by checking the evolution of the out-of-bag error.

### Genetic diversity and effective population size variation

We created one reference sequence per population in order to maximise the number of loci assembled. We filtered the sites with missing data by setting the *-minInd* flag in angsd to the total number of individuals in each population. The filtered dataset was then used to generate a *site allele frequency likelihood* (*saf*) file, where genotype likelihoods were computed using the SAMtools method (*-GL* = *1* flag). The folded site frequency spectrum (SFS) was directly computed from the filtered *saf* datasets through the realSFS program [57]. Nucleotide diversity (θ_π_), Watterson’s theta based on segregating sites (θ_w_;[61]) and Tajima’s *D* (*TD;* [62]) were computed with custom script from the SFS. Significance of *TD* was evaluated after 1000 coalescent simulations of a constant population model with size θ_w_. We reconstructed the variation in the effective population size (*N*_*e*_) through time by running the stairwayplot v.0.2 software [45] with singletons, where the composite likelihood is evaluated as the difference between the observed (folded) SFS and its expectation under a specific demographic history.

### Population divergence and migration rate estimation

Based on the results of the previous analyses, we devised five alternative Isolation/Migration (IM) models of divergence between IP and AO regions using the composite likelihood method implemented in fastsimcoal [59]*.* We presented in Fig. 3 the model richest in parameters, the remaining four representing simplified versions nested within it. Hereafter, a brief description of the five models going from the most complex to the simplest: (a) **IM-full**: the two ocean regions with their respective modern effective population sizes, ${N}_{mo{d}_{IP}}$ and ${N}_{mo{d}_{AO}}$, diverged at ${T}_{div}$ from an ancestral population of effective population size${N}_{anc}$. Due to the stairwayplot results, we allowed the two modern effective population sizes ${N}_{mo{d}_{IP}}$ and ${N}_{mo{d}_{AO}}$ to change to ${N}_{an{c}_{IP}}$ and ${N}_{an{c}_{AO}}$ following an exponential dynamic in ${T}_{{s}_{IP}}$ and ${T}_{{s}_{AO}}$ years respectively. Migration is defined by two time periods: ${m}_{1}$ representing the migration rate occurring between time 0 until ${T}_{mig}$ and ${m}_{2}$ between time ${T}_{mig}$ until${T}_{div}$. The migration matrix in each time period is asymmetric: for instance, ${m}_{{1}_{AO/IP}}$ represents the forward migration rate from AO to IP and ${m}_{{1}_{IP/AO}}$ from IP to AO. In summary, the model is defined by thirteen parameters: five effective population sizes, four migration rates and four historical events; (b) **IM-anc**: same as IM-full with ancestral migration only between ${T}_{mig}$ and${T}_{div}$. The model is defined by eleven parameters, the two ${m}_{1}$ migration rates being removed; (c) **IM-rec**: same as IM-anc, but with recent migration only occurring between time 0 until${T}_{mig}$, keeping only the two ${m}_{1}$ migration rates; (d) **IM-bsc**: the classic model where migration is constant from time 0 until ${T}_{div}$ (i.e. ${\mathrm{m}}_{1}={\mathrm{m}}_{2}=\mathrm{m}$ [28]). We modelled the variation in effective size of the two regions similarly to the other models, for a total of ten parameters; (e) **IM-div**: a pure divergence model with no migration. This is defined by eight parameters: the five effective population sizes and three historical events (${T}_{div},{T}_{{s}_{IP}}$, ${T}_{{s}_{AO}}$). The analyses are based on the folded 2D-SFS computed by angsd between six individuals from Brazil (representing the AO) and six from the Indo-Pacific (IP). This sample size was chosen to obtain a balanced design and to maximise the number of SNPs shared among the two ocean basins. Similarly, in each basin, we selected the individuals presenting the smaller proportion of missing data to further increase the number of joint SNPs. To maximize the observed 2D-SFS we applied the following options in fastsimcoal: -N 300,000 (number of coalescent simulations), -L 40 (number of expectation–maximization (EM) cycles), and -C 10 (minimum observed SFS entry count considered for parameter estimation). For all model parameters we used wide search ranges with uniform distributions (Table 2). We ran each model 100 times in order to determine the maximum likelihood parameters and to perform model selection using the Akaike’s information criterion comparing the best run of each model [59]*.* To check the robustness of the model selection procedure and to take into account the presence of linked sites in our dataset, we further examined the likelihood distribution obtained based on 100 expected 2D-SFS simulated under the parameters estimated in the best run of each model, each approximated with 10^6^ coalescent simulations. This procedure is needed to take into account the variance in the likelihood estimation given our dataset: if the distributions obtained by the various models do overlap, the difference in the estimated likelihoods of our models is not significant [63]. Finally, we determined the confidence interval of the parameter estimated under the best run of our best model by parametric bootstrapping. The 2D-SFS was bootstrapped 100 times using fastsimcoal and each of these datasets was analysed under the same conditions as the original data (one hundred independent runs for each dataset). Calibrating the molecular clock is crucial to obtain accurate estimates of demographic parameters and historical events, but it is challenging when fossil records and/or orthologous loci from an outgroup are lacking. Here, all demographic inferences were performed using the RAD-seq mutation rate of μ = 1.93 × 10^–8^ per site and per generation previously used for the tiger shark [7], and the generation time was set to 10 years [24, 64].

## Supplementary Information

**Additional file 1: Figure S1.** Isolation by distance (IBD) plot within the Indo-Pacific. Pairwise genetic distances (*F*_*ST*_/(1-*F*_*ST*_)) are plotted against geographic distances between Indo-Pacific sampling sites. **Figure S2.** Principal Component Analysis (PCA) computed with: (A) all individuals (n = 50) and (B) Indo-Pacific individuals only (n = 43). The axes represented in both panels are the first and the third component. **Figure S3.** Ancestry proportions retrieved using the *nmf* algorithm with K = 2 ancestral populations for Indo-Pacific samples performed with PCAngsd. **Figure S4.** Evolutionary scenarios used to investigate the population structure of the Atlantic Ocean based on data from Brazil population through an Approximate Bayesian Computation (ABC) framework. NS (No Structure) is an unstructured model where the modern effective size (${\mathrm{N}}_{\mathrm{mod}}$) instantaneously changes to ${\mathrm{N}}_{\mathrm{anc}}$, at time shift ${\mathrm{T}}_{\mathrm{s}}$ generations. FIM (Finite Island Meta-population) represents a finite island meta-population model with 100 demes that have been instantaneously colonised ${\mathrm{T}}_{\mathrm{col}}$ generations ago, from an ancestral population of size ${\mathrm{N}}_{\mathrm{anc}}$. Demes are allowed to exchange migrants with any other. SS (Stepping-Stone) is similar to FIM but the migrants are only exchanged between the four nearest neighbours in a two-dimensional grid. **Figure S5.** Akaike Information Criterion (AIC) values for the five isolation/migration models and the associated ranking on the x-axis. Boxplots represent the likelihood distribution of the data evaluated under the best parameter estimates for each of the five models (presented in Fig. 2) after 100 replicates. The models are presented from the richest in parameters (IM-full, 13 parameters) to the poorest (IM-div, 8 parameters). **Figure S6.** Maximum likelihood for the parameter estimated by fastsimcoal under model IM-bsc, representing two populations from each ocean basin with an effective size that changed ${\mathrm{T}}_{{\mathrm{s}}_{\mathrm{IP}}}$ and ${\mathrm{T}}_{{\mathrm{s}}_{\mathrm{AO}}}$ years ago from a modern effective size (${\mathrm{N}}_{{\mathrm{mod}}_{\mathrm{IP}}}$ and ${\mathrm{N}}_{{\mathrm{mod}}_{\mathrm{AO}}}$) to an ancestral effective size (${\mathrm{N}}_{{\mathrm{anc}}_{\mathrm{IP}}}$ and ${\mathrm{N}}_{{\mathrm{anc}}_{\mathrm{AO}}}$). The two populations are connected by an asymmetrical number of migrants constant from 0 to ${\mathrm{T}}_{\mathrm{div}}$ (${\mathrm{Nm}}_{\mathrm{IP}\to \mathrm{AO}}$ and ${\mathrm{Nm}}_{\mathrm{AO}\to \mathrm{IP}}$) and diverged ${\mathrm{T}}_{\mathrm{div}}$ years ago from an ancestral population of size ${\mathrm{N}}_{\mathrm{anc}}$.

## Data Availability

Fastq sequence files are available from the GenBank at the National Center for Biotechnology Information short-read archive database (BioProject ID: PRJNA887936).
