# Supplementary material for: Ecological and biogeographic features shaped the complex evolutionary history of an iconic apex predator (Galeocerdo cuvier)
Source: BMC Ecol Evol. 2022 Dec 16;22:147. doi: 10.1186/s12862-022-02100-y (PMC9758794; doi:10.1186/s12862-022-02100-y)
Supplement: Supplementary file 1 — Additional file 1: Figure S1. Isolation by distance (IBD) plot within the Indo-Pacific. Pairwise genetic distances (FST/(1-FST)) are plotted against geographic distances between Indo-Pacific sampling sites. Figure S2. Principal Component Analysis (PCA) computed with: (A) all individuals (n = 50) and (B) Indo-Pacific individuals only (n = 43). The axes represented in both panels are the first and the third component. Figure S3. Ancestry proportions retrieved using the nmf algorithm with K = 2 ancestral populations for Indo-Pacific samples performed with PCAngsd. Figure S4. Evolutionary scenarios used to investigate the population structure of the Atlantic Ocean based on data from Brazil population through an Approximate Bayesian Computation (ABC) framework. NS (No Structure) is an unstructured model where the modern effective size (${\mathrm{N}}_{\mathrm{mod}}$Nmod) instantaneously changes to ${\mathrm{N}}_{\mathrm{anc}}$Nanc, at time shift ${\mathrm{T}}_{\mathrm{s}}$Ts generations. FIM (Finite Island Meta-population) represents a finite island meta-population model with 100 demes that have been instantaneously colonised [file 12862_2022_2100_MOESM1_ESM.pdf]

Additional information for:

Ecological and biogeographic features shaped the complex evolutionary history of an iconic apex predator (*Galeocerdo cuvier*).

Pierre Lesturgie[1], Hugo Lainé[1], Arnaud Suwalski[1,2], Pascaline Chifflet-Belle[1,2], Pierpaolo Maisano Delser[3], Eric Clua[2,4], Sébastien Jaquemet[5], Hélène Magalon[4,5,#], Stefano Mona[1,2,4,#,*]

[1] Institut de Systématique, Evolution, Biodiversité, ISYEB (UMR 7205), Muséum National d'Histoire Naturelle, CNRS, Sorbonne Université, EPHE, Université des Antilles, Paris, France
[2] EPHE, PSL Research University, Paris, France
[3] Department of Zoology, University of Cambridge, Cambridge, UK
[4] Laboratoire d'Excellence CORAIL, Papetoai, French Polynesia
[5] UMR ENTROPIE (Université de La Réunion/IRD/CNRS), Université de La Réunion, Saint Denis, France
[#] Jointly supervised this work
* Corresponding author. E-mail: stefano.mona@mnhn.fr

Additional tables

Table S1. Matrix of pairwise F_{ST} values (lower triangle) and significancy (upper triangle). F_{ST} values in bold are significantly different from 0 ($P \leq 0.001$).

	BRA	RUN	AUS_N	COR	AUS_E	NCA
BRA		$P \leq 0.001$	$P \leq 0.001$	$P \leq 0.001$	$P \leq 0.001$	$P \leq 0.001$
RUN	**0.12**		NS[1]	NS	NS	$P \leq 0.001$
AUS_N	**0.12**	0.02		NS	NS	NS
COR	**0.13**	0.03	0.02		NS	NS
AUS_E	**0.12**	0.02	0.02	0.02		$P \leq 0.001$
NCA	**0.12**	**0.03**	0.02	0.02	**0.03**	

[1]NS: Not Significant

Table S2. Prior distribution of the parameters of the Finite Island (FIM), Stepping Stone model (SS) and Non-Structured (NS) models. *Nm* represents the number of migrants exchanged per generation either with the four closest neighbouring demes (SS) or with any deme in the matrix (FIM). N_{mod} represents the modern effective population size of the NS model. N_{anc} represents the ancestral effective population size either of the founding deme (in the structured models) or in the panmictic population (NS model). T_{col} is the colonization time of the array of deme (FIM and SS only) and T_c is the time when a change in effective population size happened in the panmictic population (NS only). Time parameters are in generations.

FIM	Nm^*<br>P*: 0.001 - 100	T_{col}§<br>U¶: 1 – 300,000	N_{anc}<br>U: 100 – 100,000
SS	Nm^*<br>P: 0.001 - 100	T_{col}§<br>U: 1 – 300,000	N_{anc}<br>U: 100 – 100,000
NS	N_{mod}<br>U: 1 – 100,000	T_s§<br>U: 1 – 300,000	N_{anc}<br>U: 1 – 100,000

* P: the prior distribution of *Nm* is the product of two uniforms (one for *N* and one for *m*).

¶ U: uniform distribution.

Table S3. Confusion matrix of the model selection procedure and posterior probability for the most likely model explaining the structuring: rows indicate the simulated models and columns the votes (in %) attributed by the ABC-RF algorithm to each of them.

		Attributed votes (%)			Class. error	Posterior Probability
		FIM	NS	SS		
BRA	FIM	75.848	4.438	19.714	0.24152	0.63
	NS	1.466	97.158	1.376	0.02842	
	SS	20.584	4.088	75.328	0.24672	
RUN	FIM	44885	1376	3739	0.1023	0.79
	NS	466	49095	439	0.0181	
	SS	4443	781	44776	0.10448	
AUS_N	FIM	40806	1867	7327	0.18388	0.48
	NS	598	48738	664	0.02524	
	SS	7591	1466	40943	0.18114	
COR	FIM	37878	2121	10001	0.24244	0.69
	NS	755	48450	795	0.031	
	SS	10344	1917	37739	0.24522	
AUS_E	FIM	40162	1849	7989	0.19676	0.86
	NS	623	48720	657	0.0256	
	SS	8334	1562	40104	0.19792	
NCA	FIM	42620	1584	5796	0.1476	0.89
	NS	543	48872	585	0.02256	
	SS	6123	1184	42693	0.14614	

Additional figures

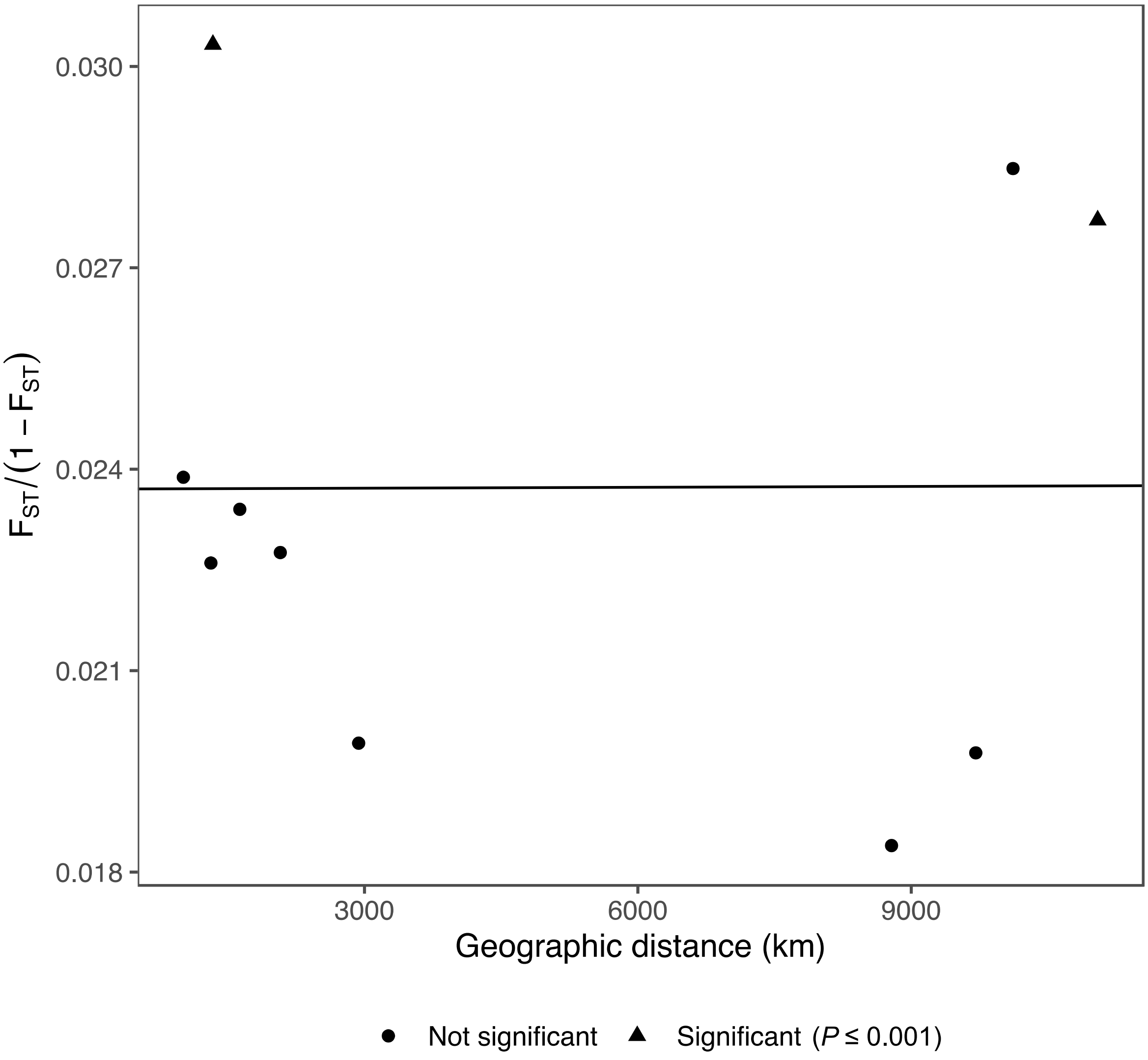

Figure S1. Isolation by distance (IBD) plot within the Indo-Pacific. Pairwise genetic distances (F_{ST}/(1-F_{ST})) are plotted against geographic distances between Indo-Pacific sampling sites.

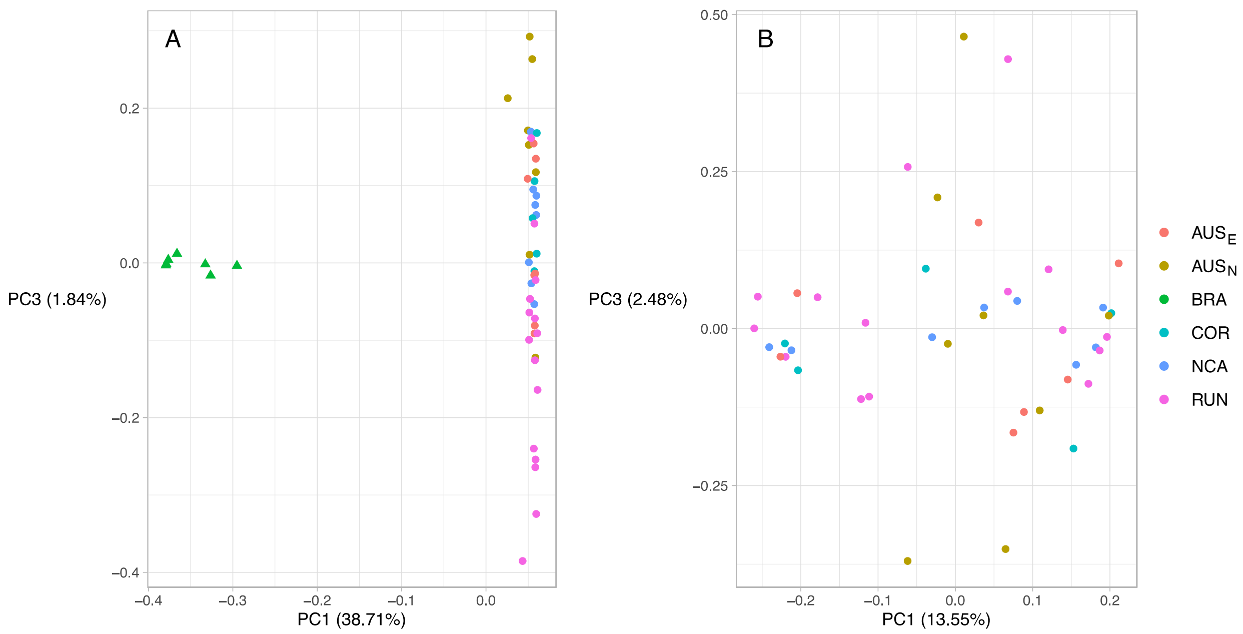

Figure S2. Principal Component Analysis (PCA) computed with: (A) all individuals (n = 50) and (B) Indo-Pacific individuals only (n = 43). The axes represented in both panels are the first and the third component.

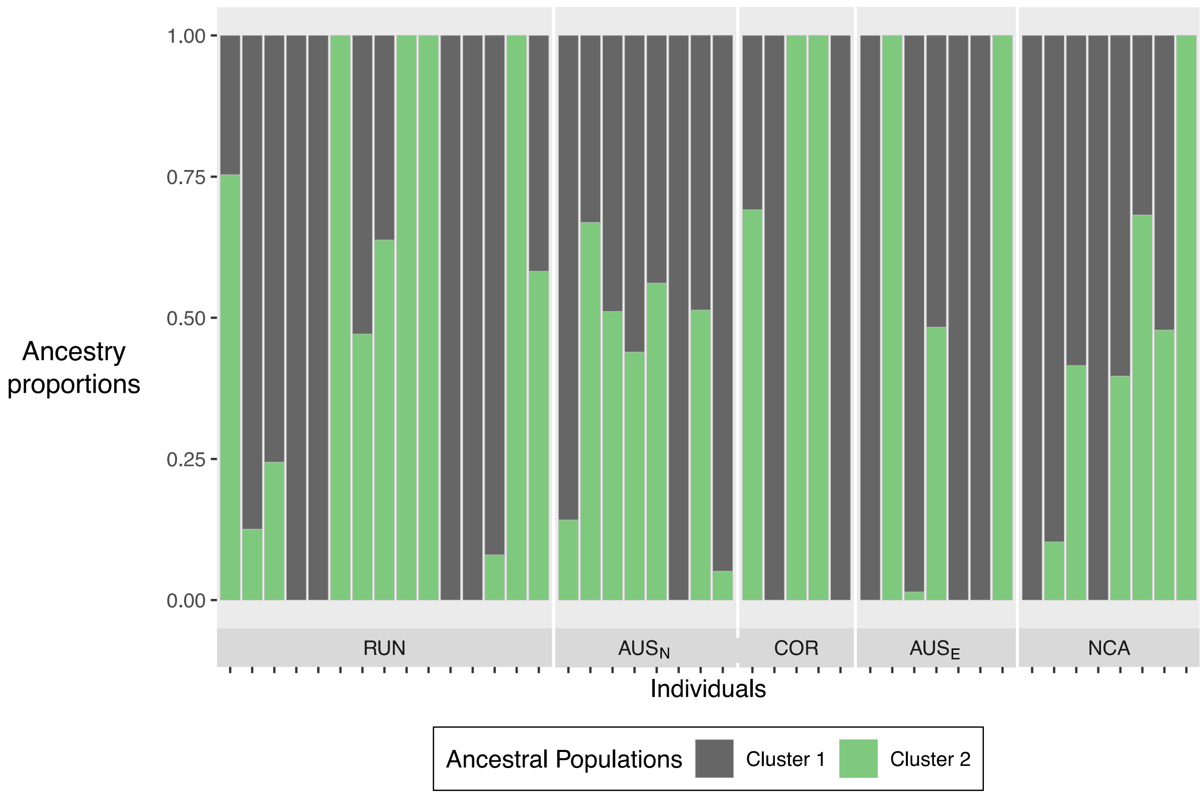

Figure S3. Ancestry proportions retrieved using the *nmf* algorithm with K=2 ancestral populations for Indo-Pacific samples performed with PCANGSD.

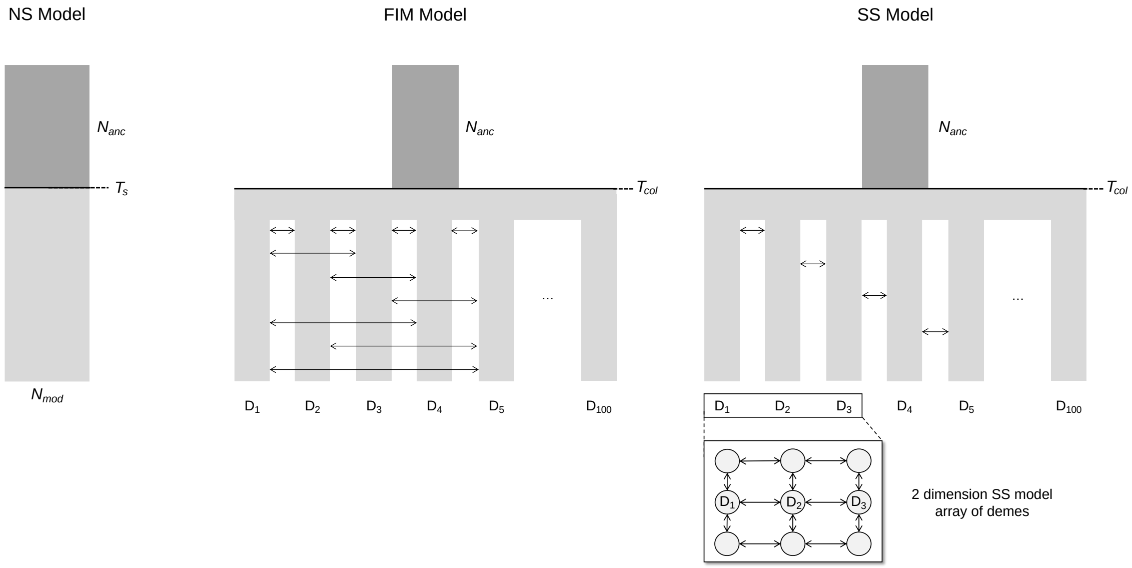

Figure S4. Evolutionary scenarios used to investigate the population structure of the Atlantic Ocean based on data from Brazil population through an Approximate Bayesian Computation (ABC) framework. NS (No Structure) is an unstructured model where the modern effective size (N_{mod}) instantaneously changes to N_{anc}, at time shift T_s generations. FIM (Finite Island Meta-population) represents a finite island meta-population model with 100 demes that have been instantaneously colonised T_{col} generations ago, from an ancestral population of size N_{anc}. Demes are allowed to exchange migrants with any other. SS (Stepping-Stone) is similar to FIM but the migrants are only exchanged between the four nearest neighbours in a two-dimensional grid.

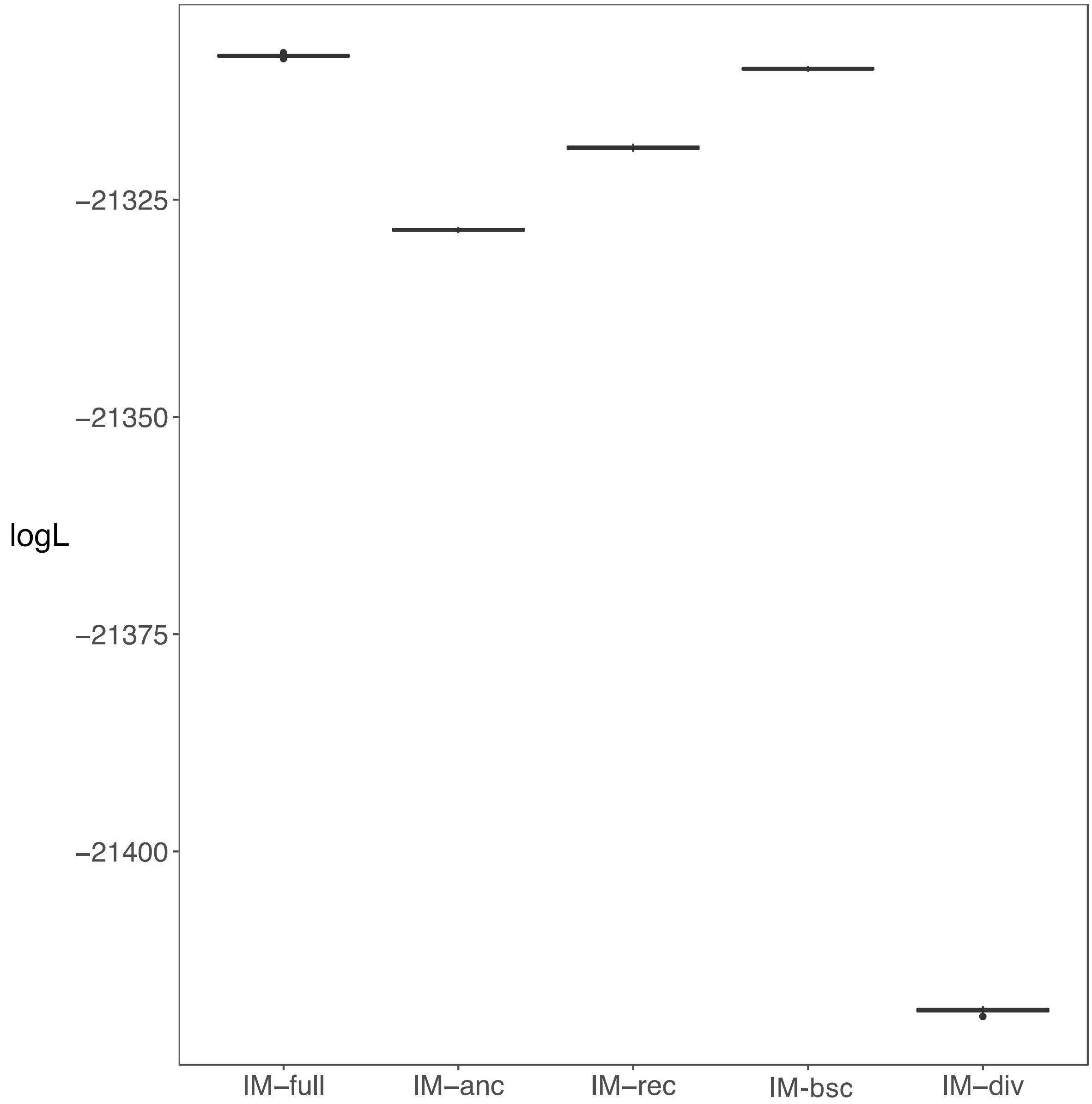

Figure S5. Akaike Information Criterion (AIC) values for the five isolation/migration models and the associated ranking on the *x*-axis. Boxplots represent the likelihood distribution of the data evaluated under the best parameter estimates for each of the five models (presented in Figure 2) after 100 replicates. The models are presented from the richest in parameters (IM-full, 13 parameters) to the poorest (IM-div, 8 parameters).

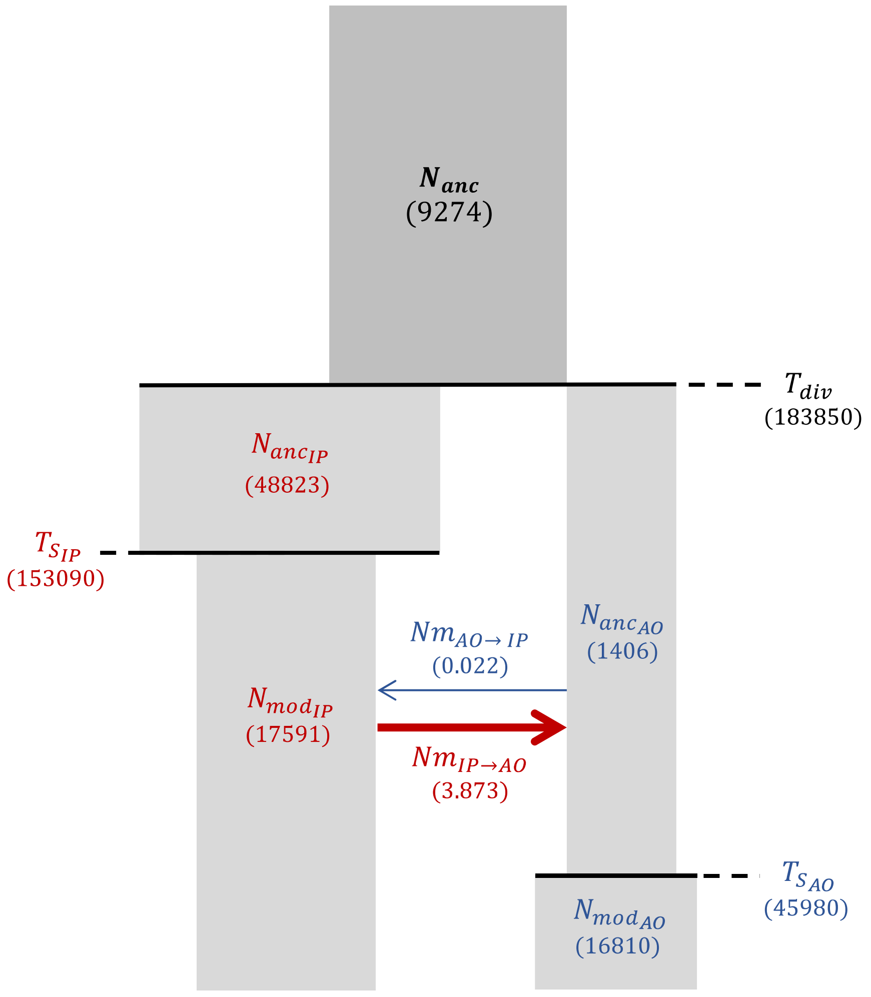

Figure S6. Maximum likelihood for the parameter estimated by fastsimcoal under model IM-bsc, representing two populations from each ocean basin with an effective size that changed $T_{s_{IP}}$ and $T_{s_{AO}}$ years ago from a modern effective size ($N_{mod_{IP}}$ and $N_{mod_{AO}}$) to an ancestral effective size ($N_{anc_{IP}}$ and $N_{anc_{AO}}$). The two populations are connected by an asymmetrical number of migrants constant from 0 to T_{div} ($Nm_{IP\rightarrow AO}$ and $Nm_{AO\rightarrow IP}$) and diverged T_{div} years ago from an ancestral population of size N_{anc}.

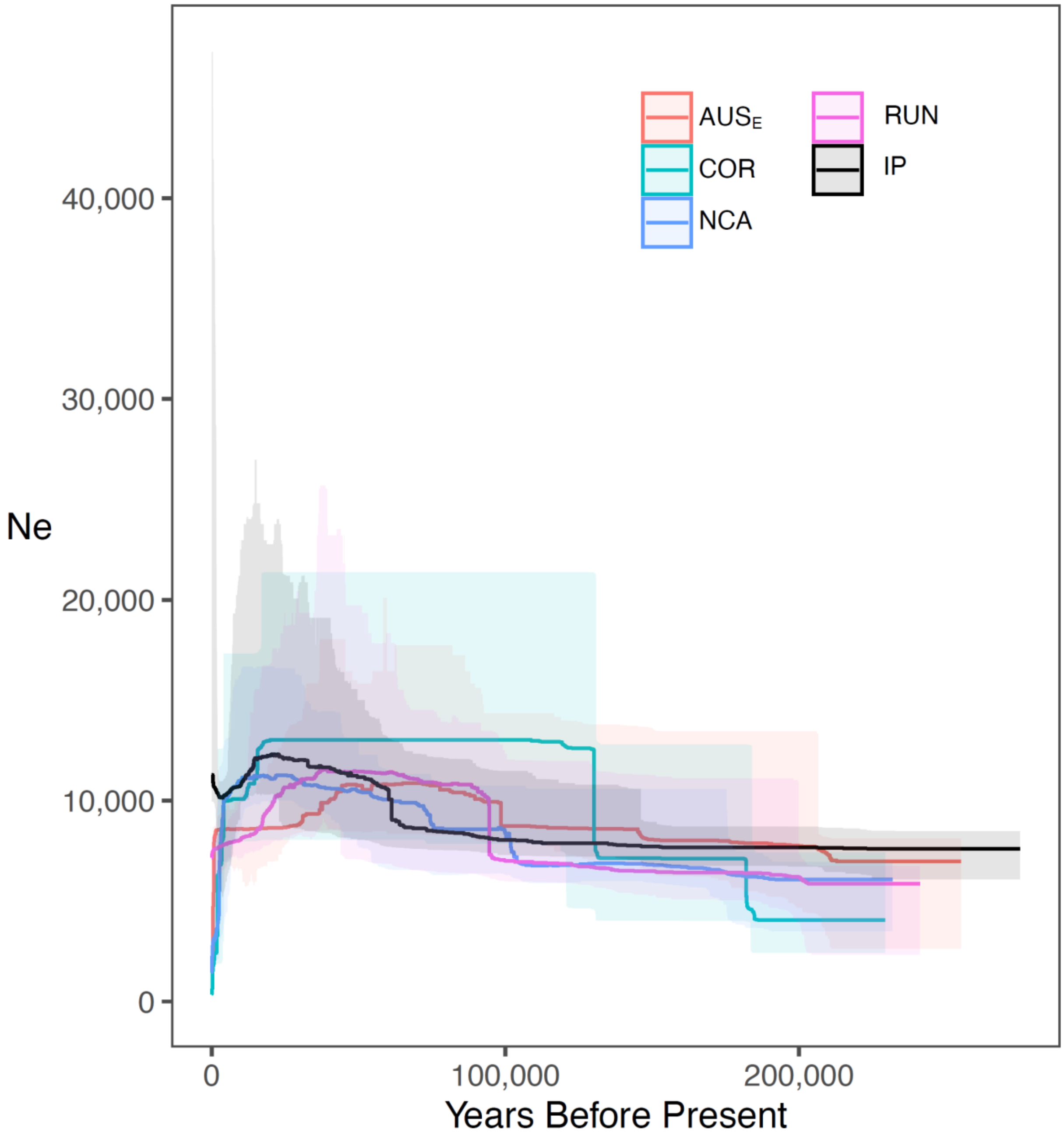

Figure S7. Variation of the effective population size (N_e) through time and its 75% confidence interval estimated by the STAIRWAYPLOT for all sampling sites. AUS$_E$: East Coast of Australia; AUS$_N$: North Coast of Australia; BRA: Brazil; COR: Coral Sea; NCA: New Caledonia; RUN: Reunion Island; IP: pooled individuals from AUS$_E$, COR, NCA and RUN sampling locations.
